# Supplementary material for: Novel 2-Thiouracil-5-Sulfonamide Derivatives: Design, Synthesis, Molecular Docking, and Biological Evaluation as Antioxidants with 15-LOX Inhibition
Source: Molecules. 2023 Feb 17;28(4):1925. doi: 10.3390/molecules28041925 (PMC9963659; doi:10.3390/molecules28041925)
Supplement: Supplementary file 1 [file molecules-28-01925-s001.zip › molecules-2215252-supplementary.pdf]

Supplementary material

For

Novel 2-Thiouracil-5-Sulfonamide Derivatives: design, synthesis, molecular docking, and biological evaluation as antioxidant with 15-LOX inhibition

Naglaa M. Ahmed [a,*] , Ahmed H. Lotfallah [b] , Mohamed S.Gaballah [c], Samir M. Awad [a] and Moustafa K. Soltan [d,e]

[a] *Pharmaceutical Organic Chemistry Department, Faculty of Pharmacy, Helwan University, Ein Helwan, P.O. Code11795, Cairo, Egypt*

[b] Department of Pharmaceutical Chemistry, Faculty of Pharmacy, Sinai University, 16020, El-Arish, Egypt.

[c] *Biochemistry Department, Faculty of Pharmacy, Helwan University, Ein Helwan, P.O. Code 11795, Cairo, Egypt*

[d] *Oman college of Health sciences, Muscat, Sultanate of Oman*

[e] *Medicinal Chemistry Department, Faculty of Pharmacy, Zagazig University, Egypt*

*Correspondence e-mail: Dr. Naglaa Ahmed nogamoon2005@yahoo.com
and Nagla_abdelal@pharm.helwan.edu.eg (N. M. Ahmed).

1-Biological Evaluation Details:

a-*In-vitro* Assays for biological antioxidant Activity

- DPPH scavenging method.

A 96-well plate was filled with 100 µL of the investigated compounds at various concentrations (12.5, 25, 50, 100, and 200 µg/mL). The plate was then incubated at room temperature for 30 min while being protected from light by adding 100 µL of 100µM DPPH methanolic solution to each well. At a λ_{517} nm. the solution's absorbance was measured. The positive control was ascorbic acid, and the negative control was DMSO. According to the following equation, the percentage of DPPH scavenging activity was determined:

% of DPPH scavenging = $[(A_{control} - A_{sample}) / A_{control}]$ x100.

where $A_{control}$ is the absorbance of the control reaction (with all reagents except the test compound), and A_{sample} is the absorbance of the test sample. Linear regression analysis was performed to calculate drug concentration showing 50% free radical inhibition activity (IC_{50}). All tests were performed in triplicates.

-Scavenging of hydrogen peroxide

In this method, the decay or loss of hydrogen peroxide can be quantified spectrophotometrically at 230 nm when a scavenger is incubated with it. The procedure was applied with a few changes. In phosphate buffered saline, hydrogen

peroxide (20 mM) solution was prepared in phosphate buffered saline (PBS, pH 7.4). In order to make different concentrations of 1 ml of the tested substances (3–9) or standards in methanol, 2 ml of hydrogen peroxide solution in PBS was added. After 10 minutes, the absorbance of H2O2 was measured at 230 nm in comparison to an ascorbic acid (AA) reference solution that contained phosphate buffer but no H2O2.

% of H_2O_2 scavenging = [(A $_{control}$ -A $_{sample}$) / A $_{control}$] x100.

where A $_{control}$ is the absorbance of the control reaction (with all reagents except the test compound), and A $_{sample}$ is the absorbance of the test sample. Linear regression analysis was performed to calculate drug concentration showing 50% free radical inhibition activity (IC_{50}). All tests were performed in triplicates.

- Lipid peroxidation assay

A mixture of 0.1 mol/L linoleic acid (0.2 mL), 2.0 mmol/L FeCl2(H2O)$_4$(0.2 mL), 2.0 mmol/L H2O2(0.2 mL), and 0.2 mol/L phosphate buffer (pH 7.4, 5 mL) , was combined with the test substances . For 24 hours, the reaction mixture was incubated at 37°C. After the mixture had been incubated, 0.2 mL of BHA (20 mg/mL), 1.0 mL of thiobarbituric acid (10 mg/mL), and 1.0 mL of trichloroacetic acid (100 mg/mL) were added. The mixture was then cooked in a boiling water bath for 30 minutes. 5.0 mL of chloroform was added after cooling, and the mixture underwent a 1000^{x}g centrifugation to produce a supernatant. At 532 nm, the supernatant's absorbance was determined spectrophotometrically. The average of three duplicate analyses represents all test data. The inhibition of $FeCl_2/H_2O_2$-stimulated linoleic acid peroxidation (%) was calculated as follows:

Inhibition of peroxidation linoleic acid(%) = [1-(ΔA $_{532,sample}$)/(ΔA $_{532,control}$)] ^{x} 100

The 50% of inhibitory concentration (IC_{50}) was measured.

Ascorbic acid was used as reference.

2-Molecular Modeling

To study the active site prior to docking, the crystal structure of protocatechuic acid with LOX (1N8Q) was superimposed with the human crystal structure of LOX (4NRE). The superimposition shows that both the co crystallized substrate mimic in 4NRE and protocatechuic acid (co crystallized in 1N8Q) occupy overlapping regions (i.e. Ile 857, Gln 514 and Trp 519 in 1N8Q correspond to Ile 676, Glu 369 and Leu 374 in 4NRE respectively, Figure .5). However, the substrate mimic in 4NRE was protruding more away from protocatechuic acid and thus interacting with further amino acids. Such extra binding is expected due to the pronounced size difference between the two ligands. Accordingly, This variation directed us to use the human crystal structure of LOX (4NRE) for docking. In this work, the region occupied by the co crystallized substrate mimic was used to set the site of docking in 4NRE. For reference, the co crystallized ligand in 4NRE was also included in the docking experiment. The protein was prepared with the AMBER12-EHT forcefield. Rigid-body docking using the default parameters and visualisation was performed with MOE, the 2020.09 version, Chemical Computing Group ULC, Montreal, QC, Canada.

3- **Representative ^{13}C , ^{1}HNMR and MASS Spectra :**

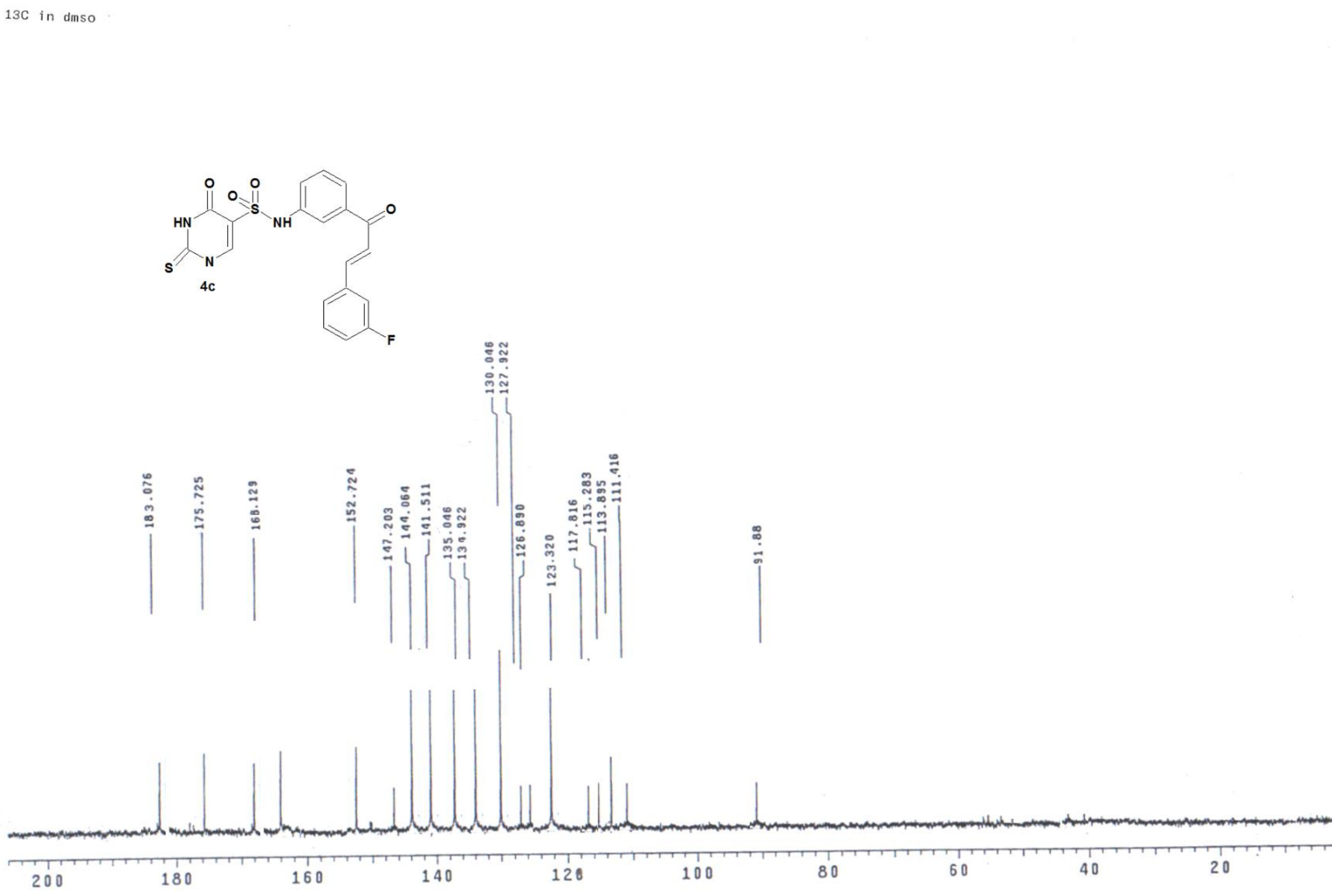

Figure S1. ^{13}C NMR spectrum of compound **4c**

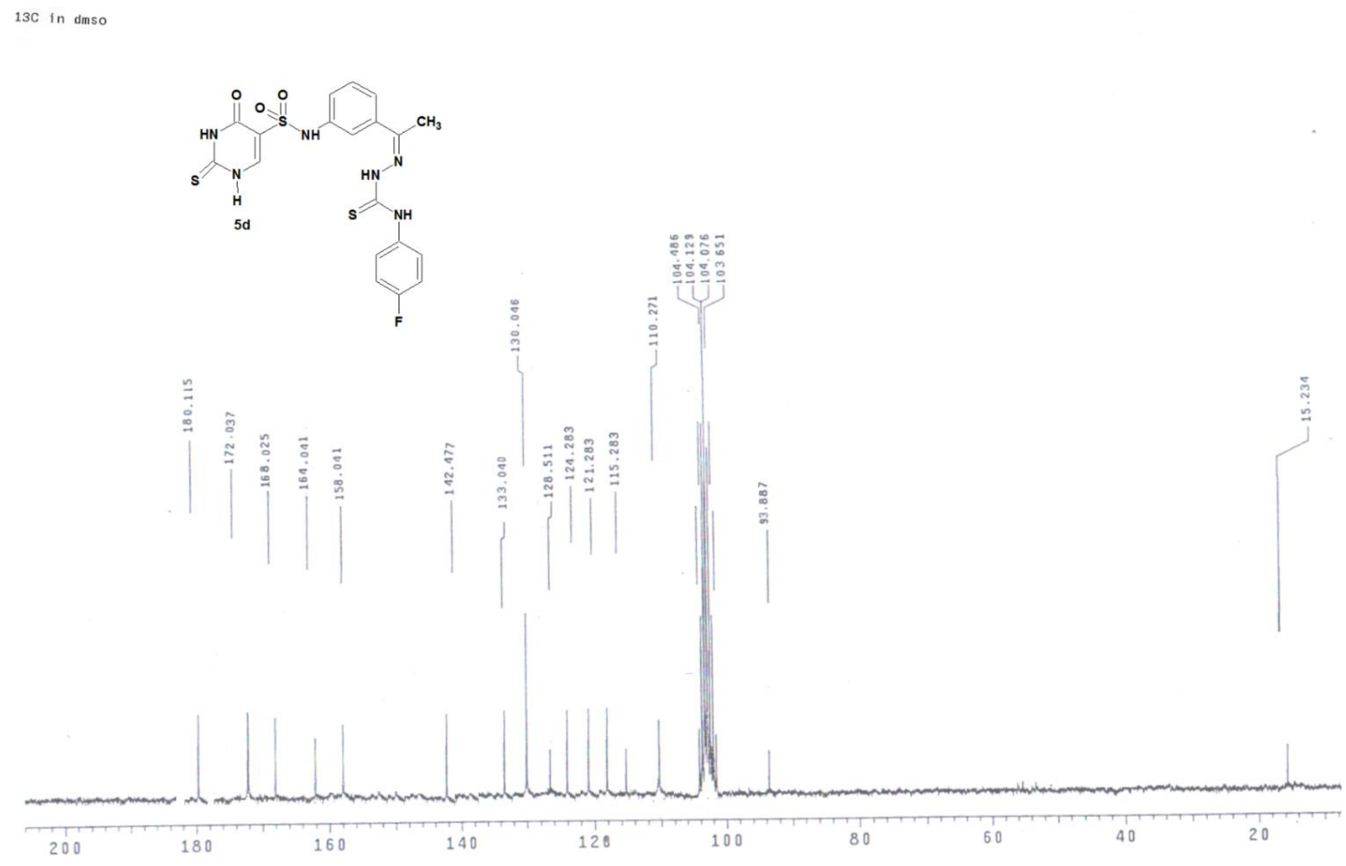

Figure S2. ^{13}C NMR spectrum of compound **5d**

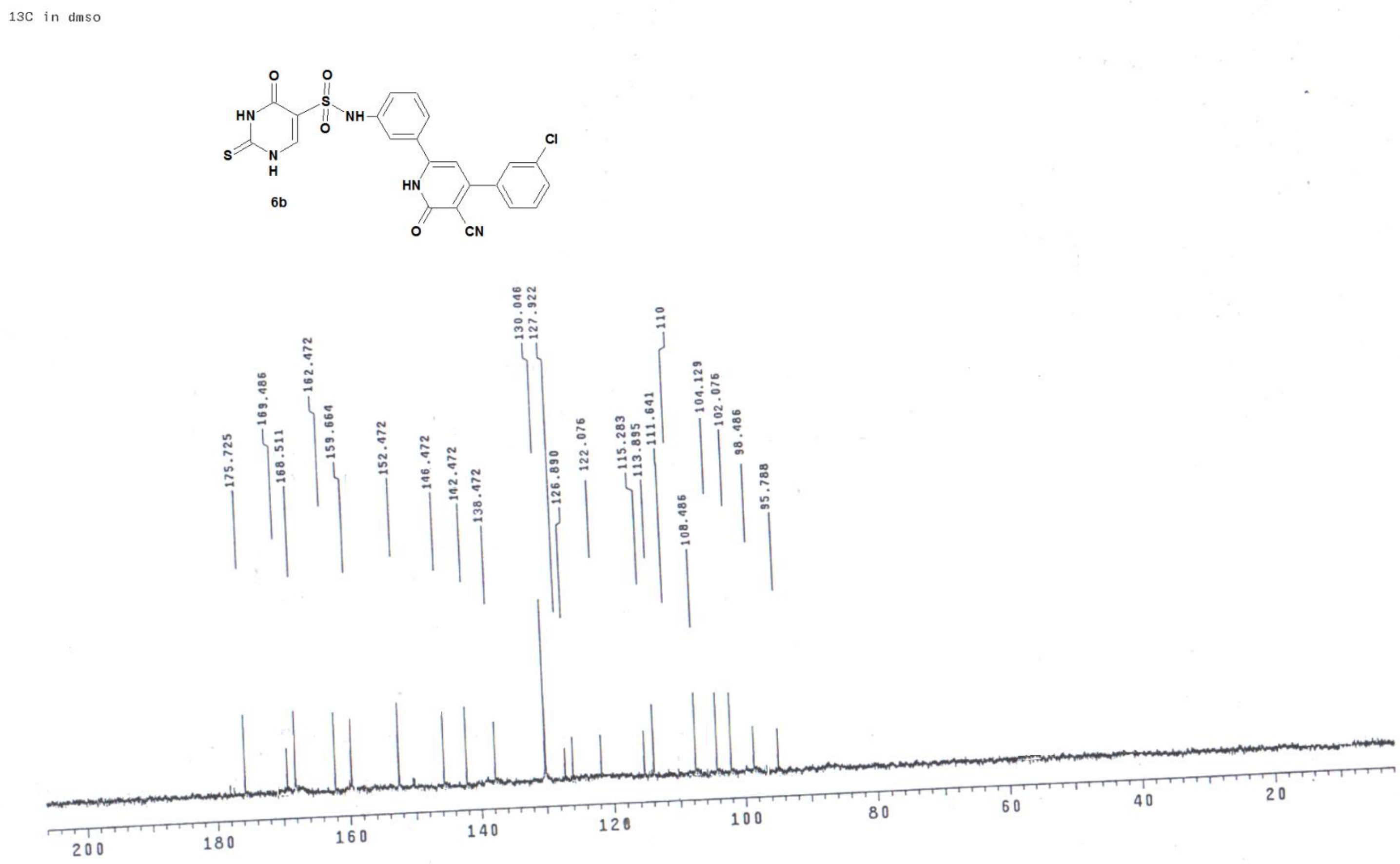

Figure S3. ^{13}C NMR spectrum of compound **6b**

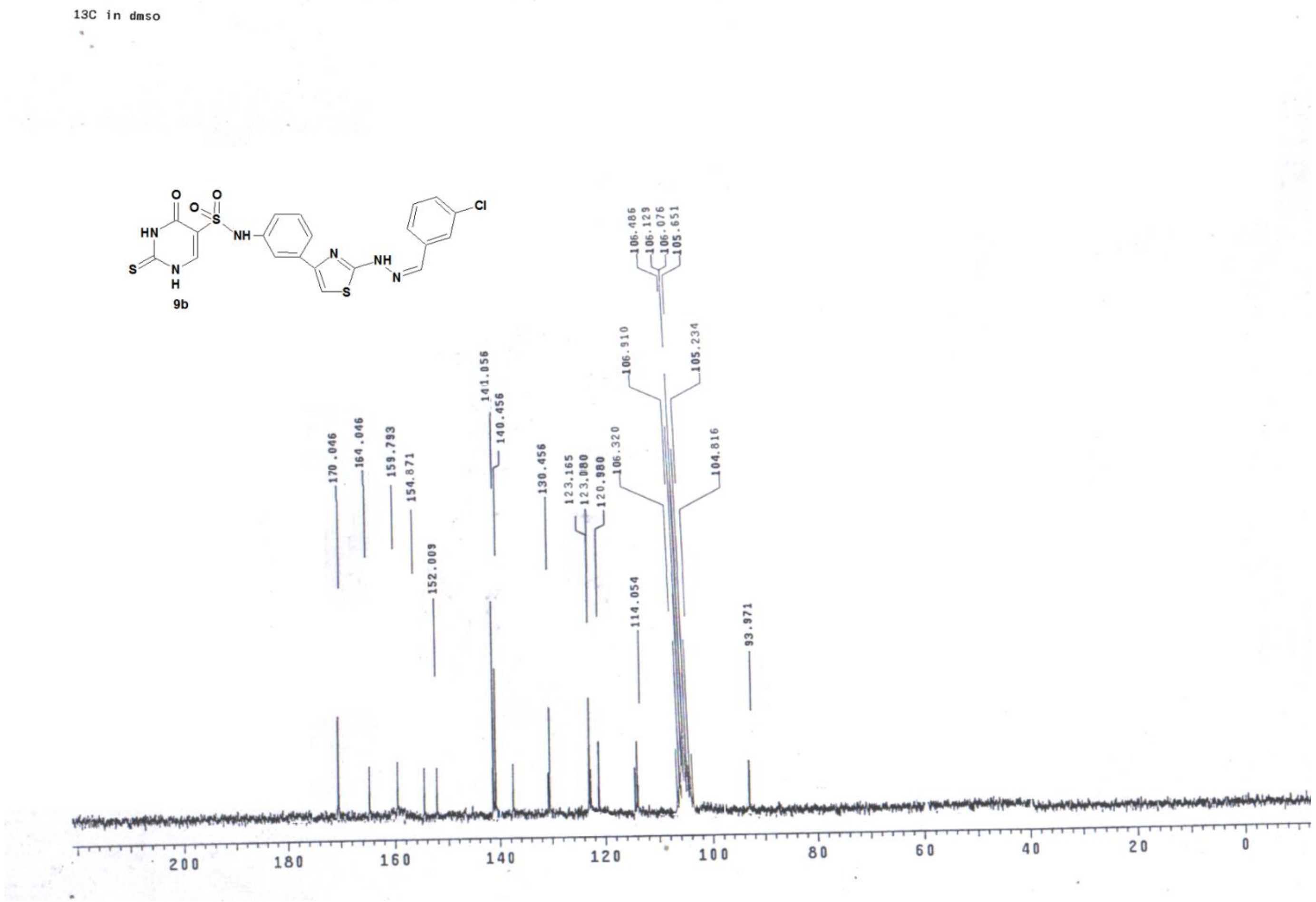

Figure S4. ^{13}C NMR spectrum of compound **9b**

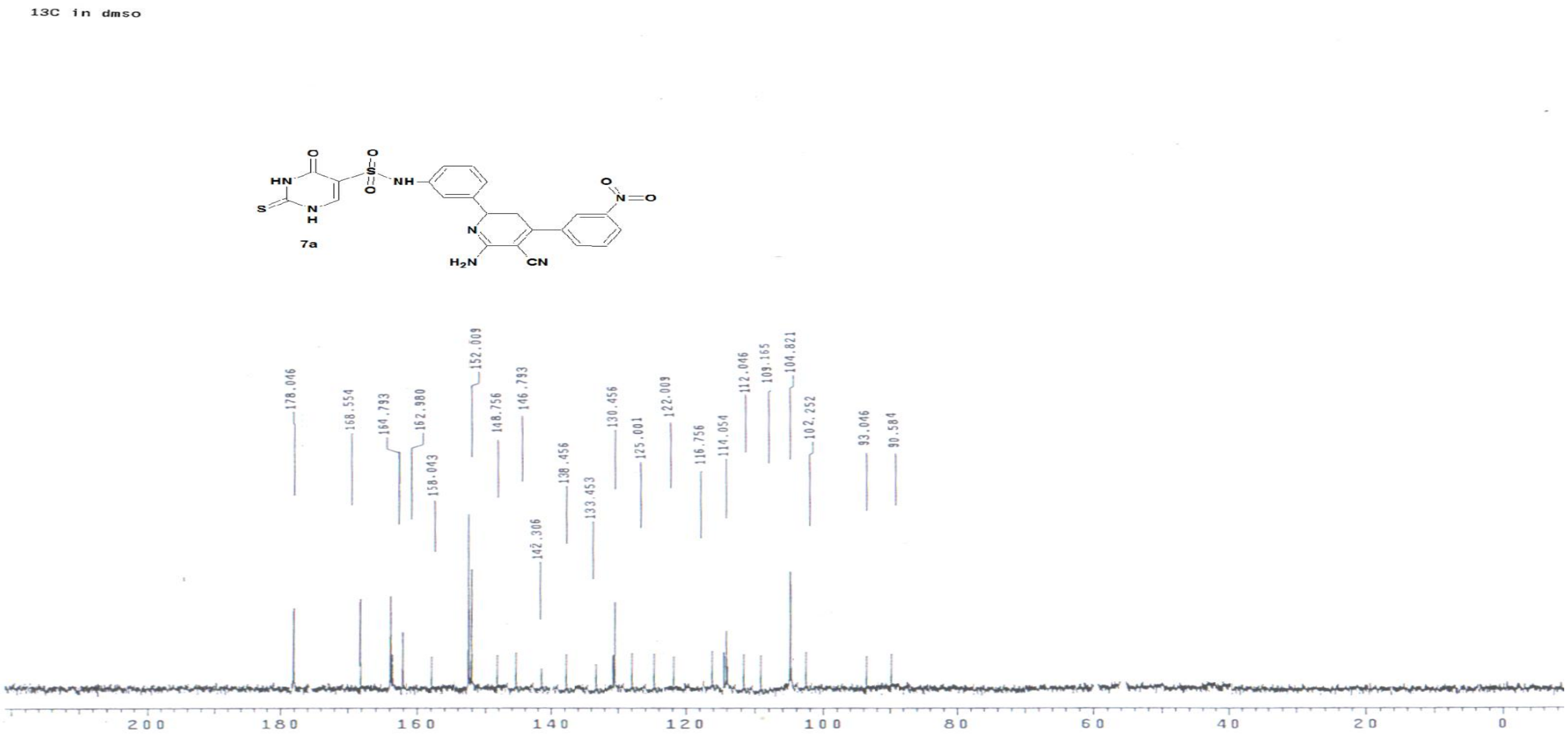

Figure S5. ^{13}C NMR spectrum of compound **7a**

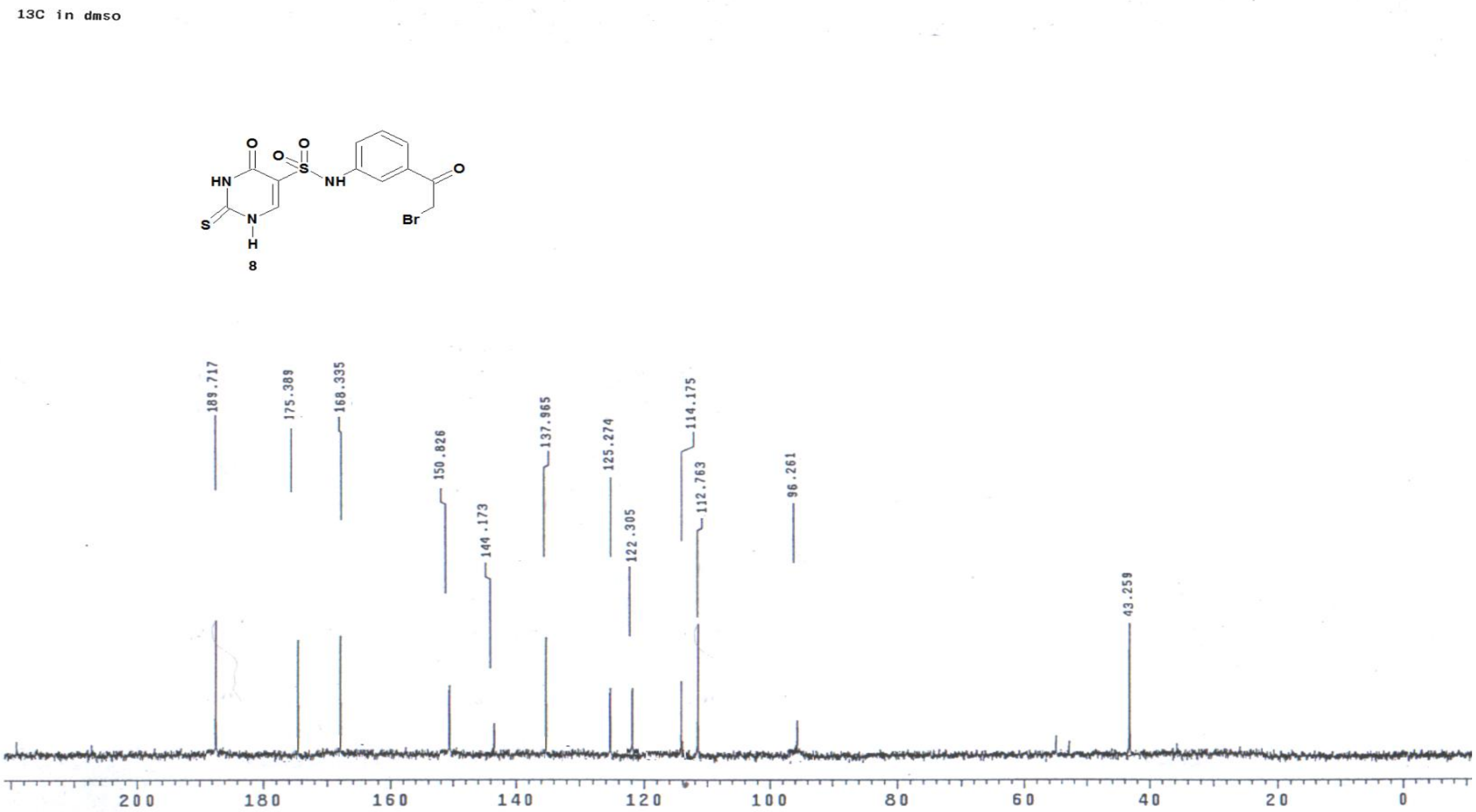

Figure S6. ^{13}C NMR spectrum of compound **8**

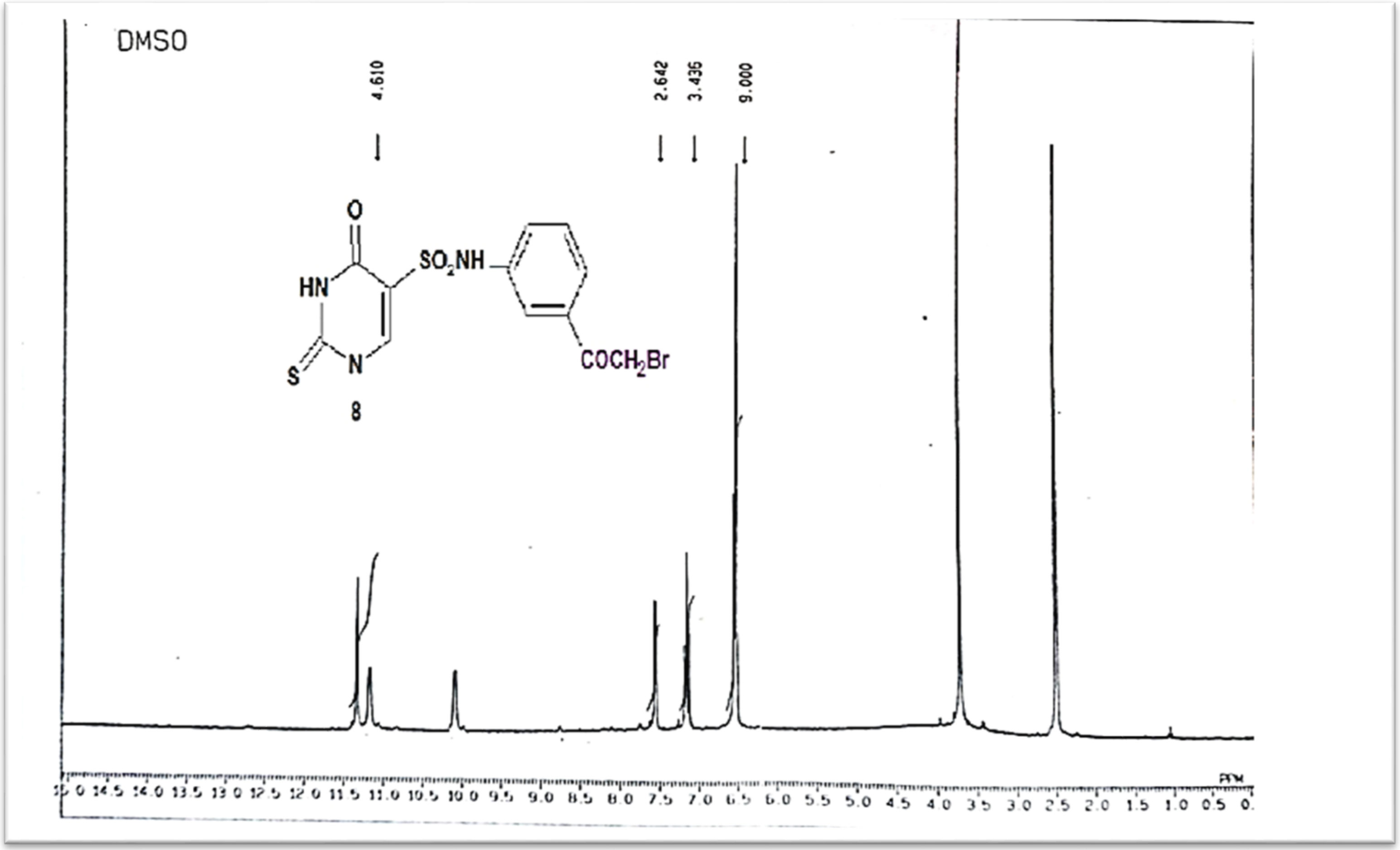

FigureS7. ^{1}H NMR spectrum of compound **8**

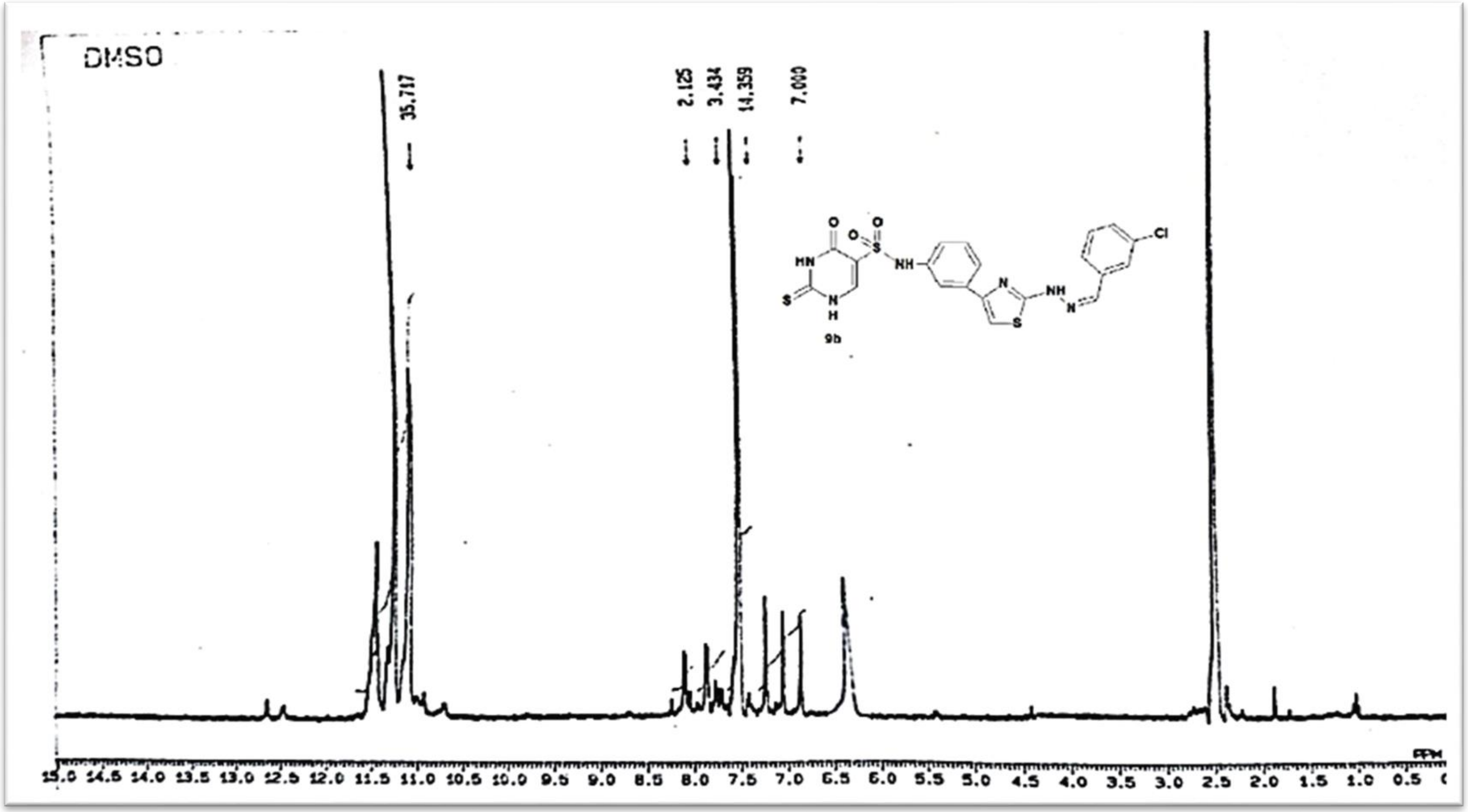

FigureS8. ^{1}H NMR spectrum of compound 9b

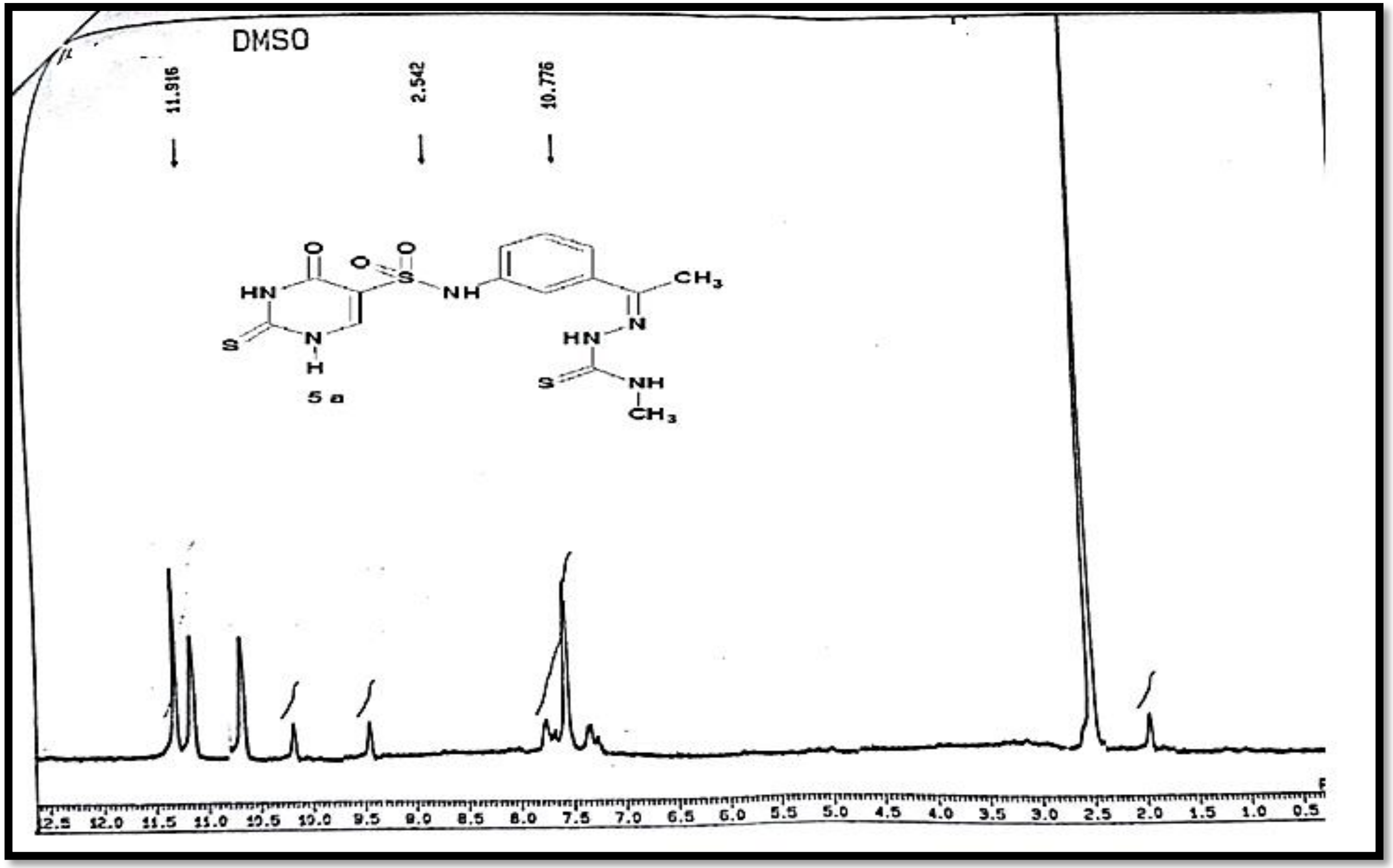

FigureS9. ^{1}H NMR spectrum of compound 5a

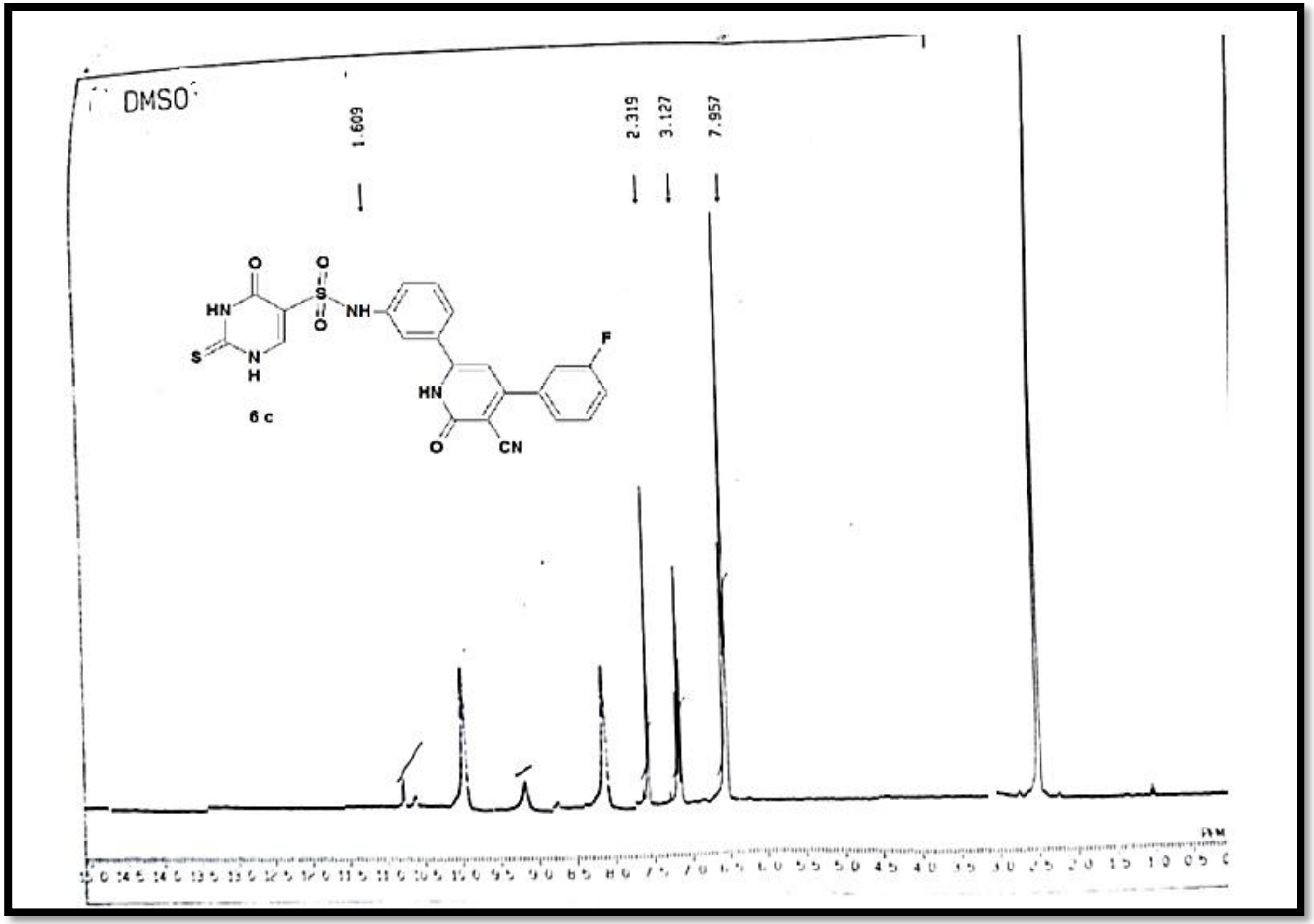

FigureS10. ^{1}H NMR spectrum of compound 6c

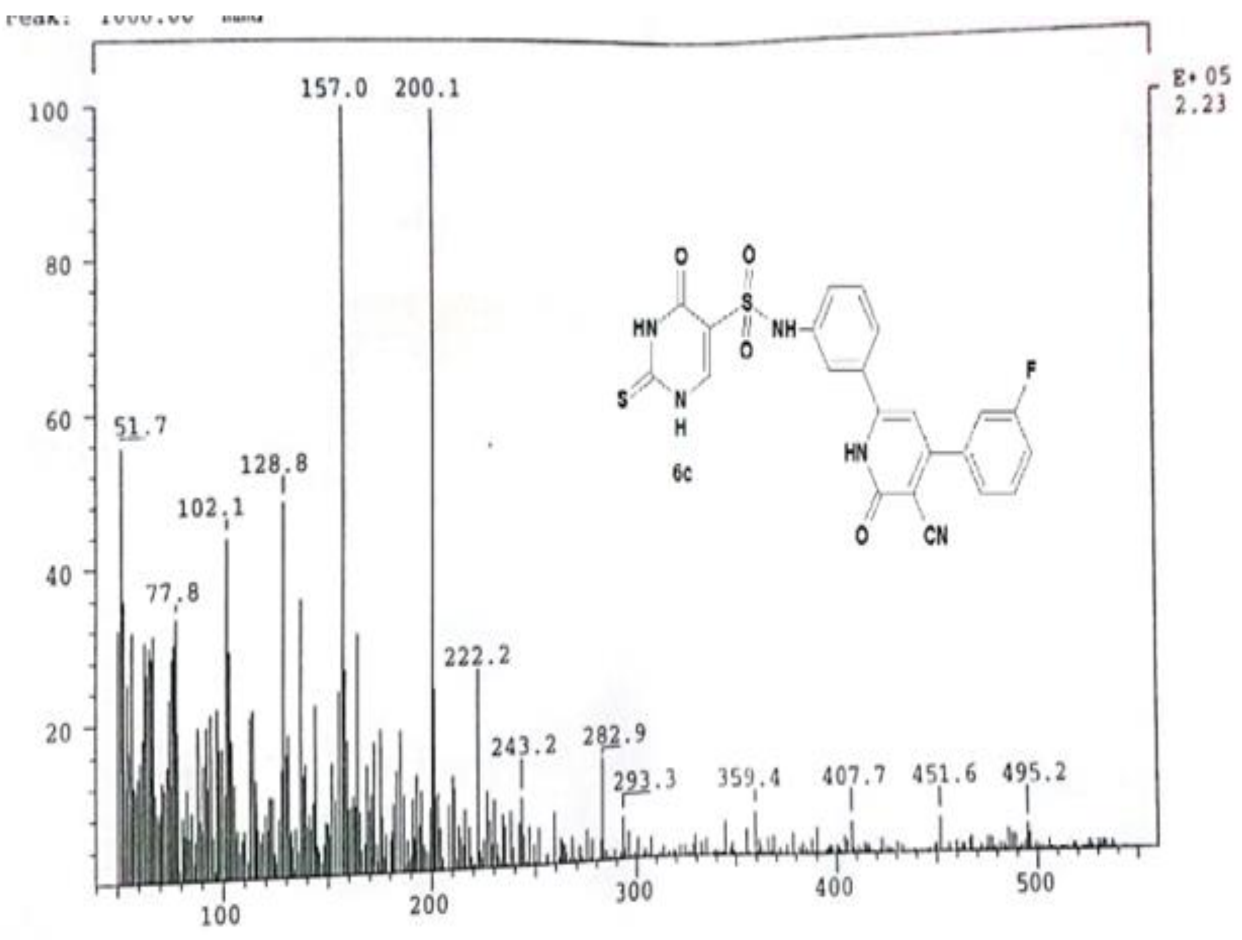

FigureS11. (EI mass) spectrum of compound 6c

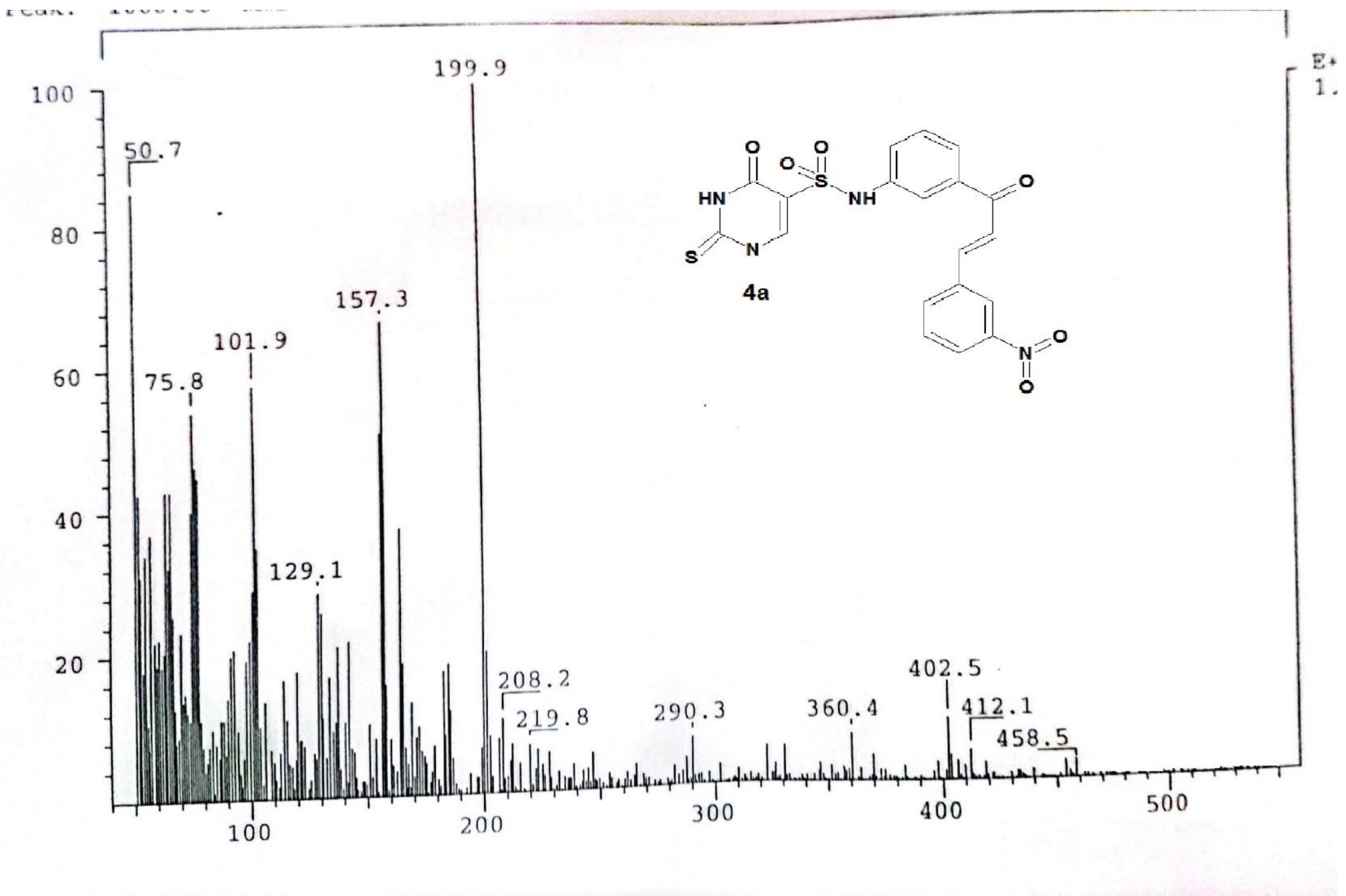

FigureS12. (EI mass) spectrum of compound 4a

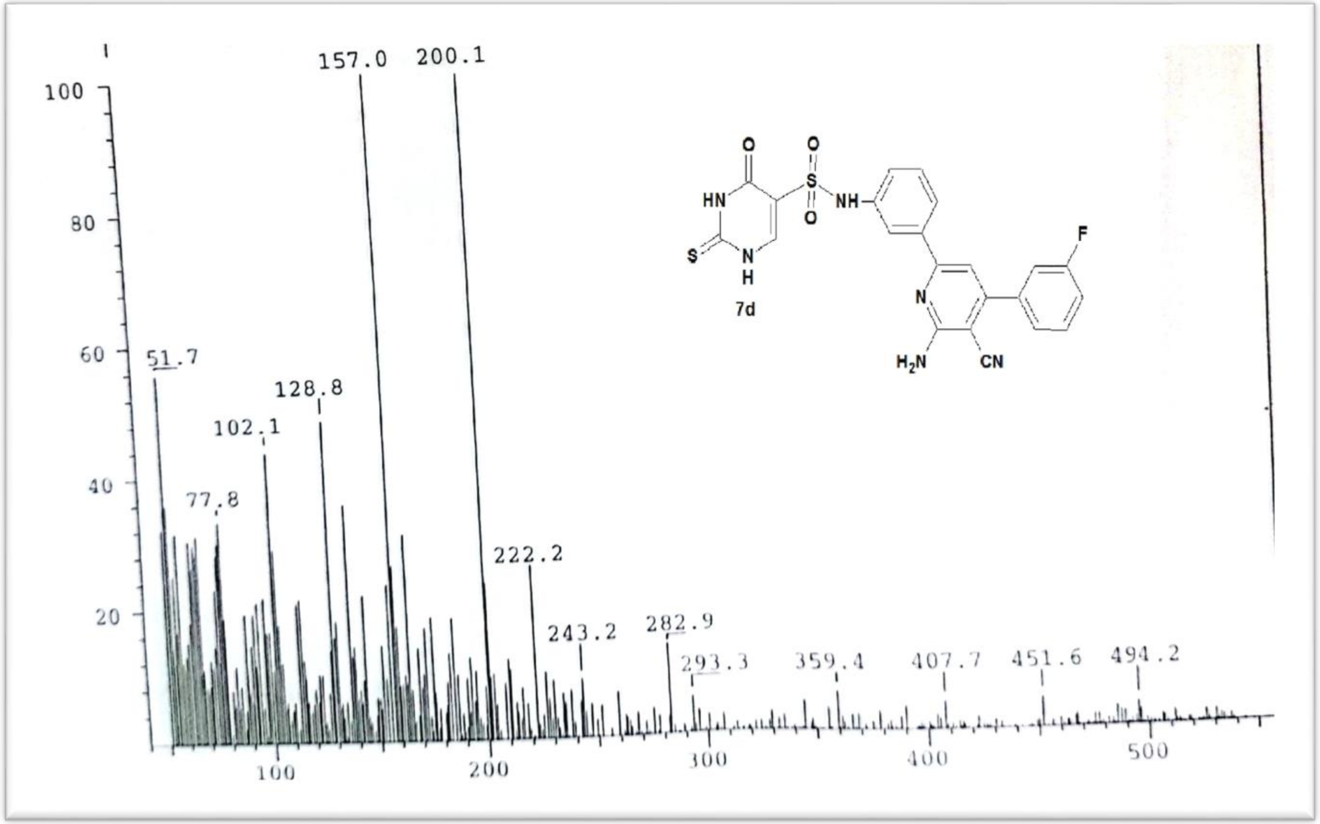

FigureS13. (EI mass) spectrum of compound 7d

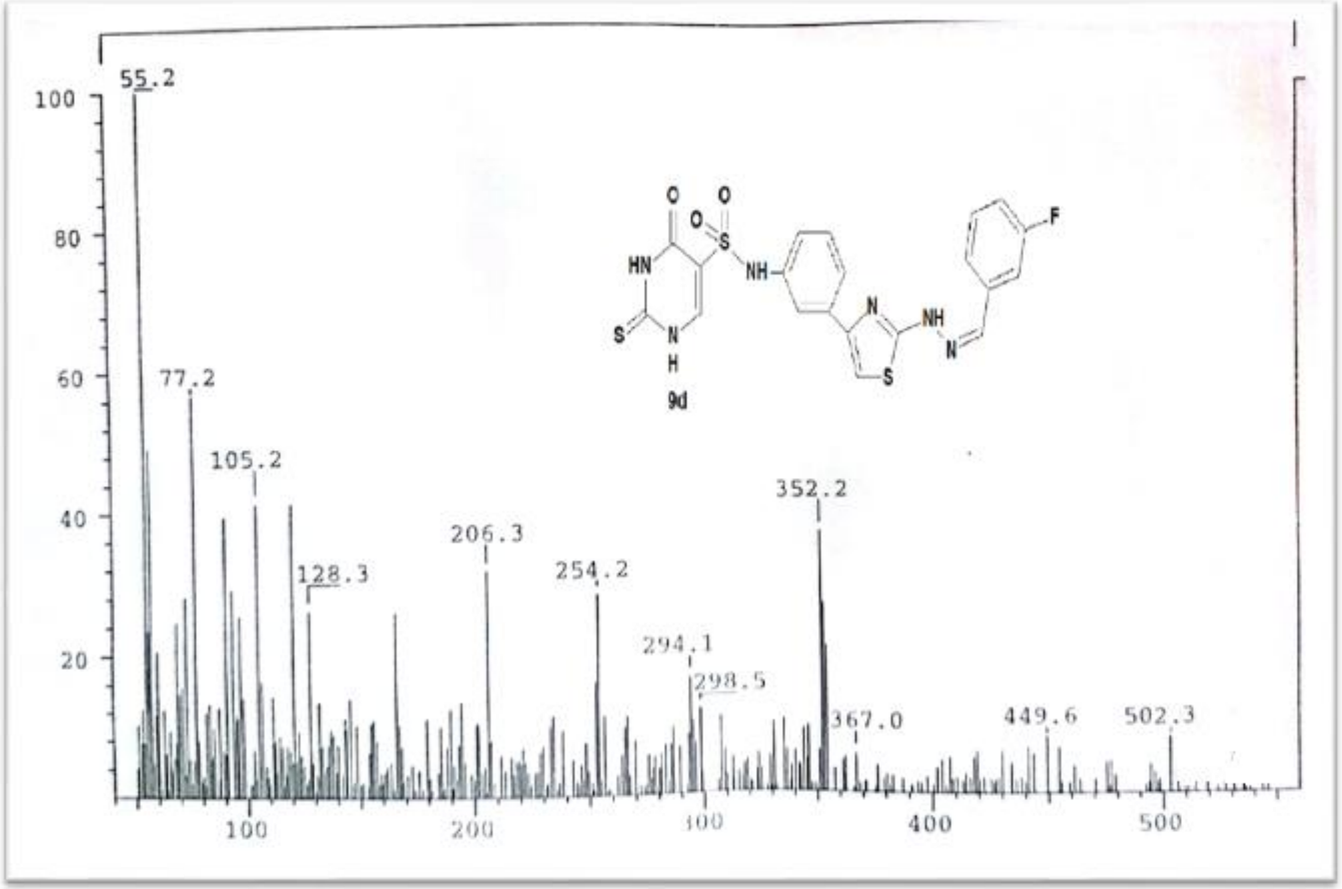

FigureS14. (EI mass) spectrum of compound 9d

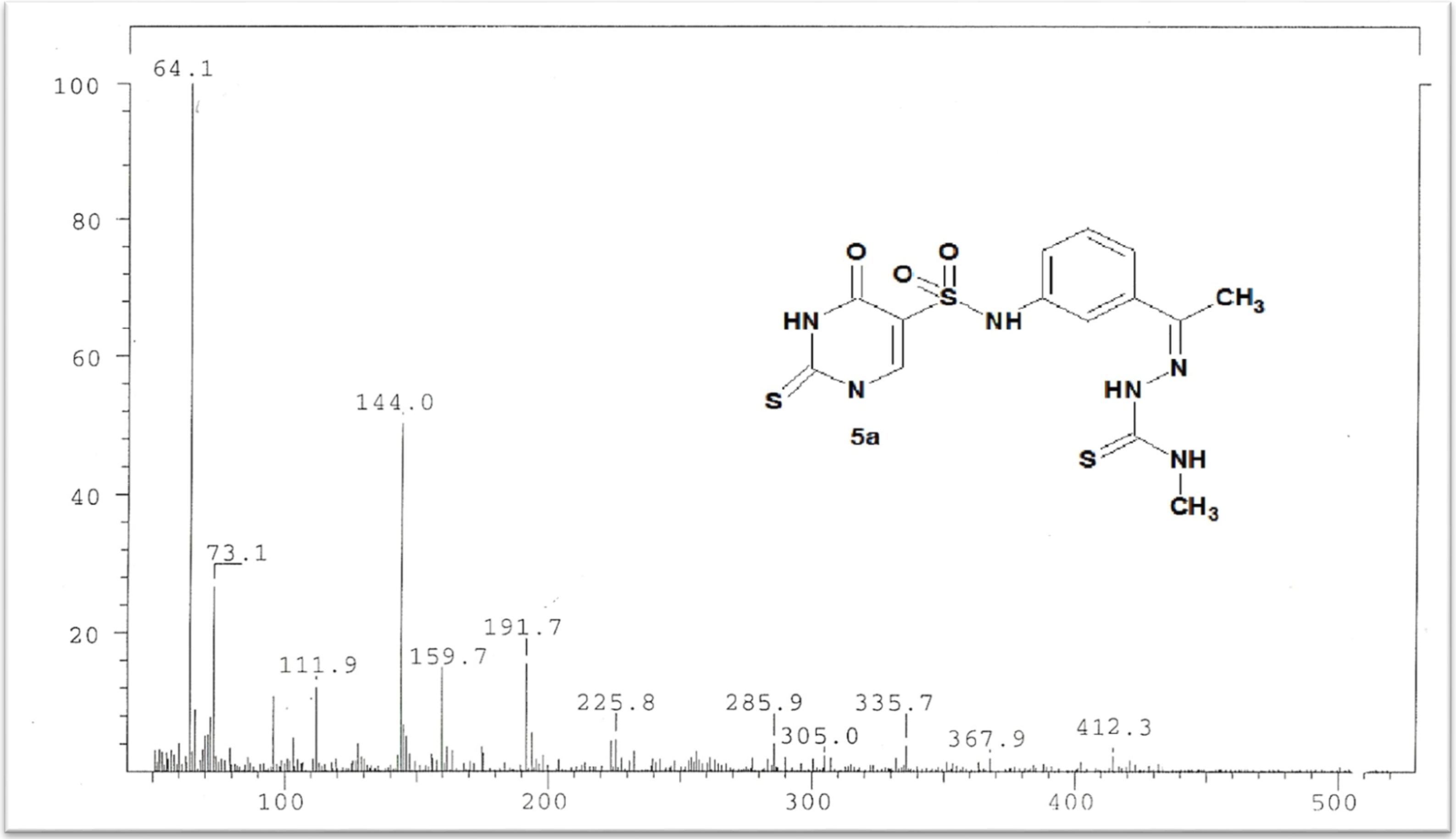

FigureS15. (EI mass) spectrum of compound 5a

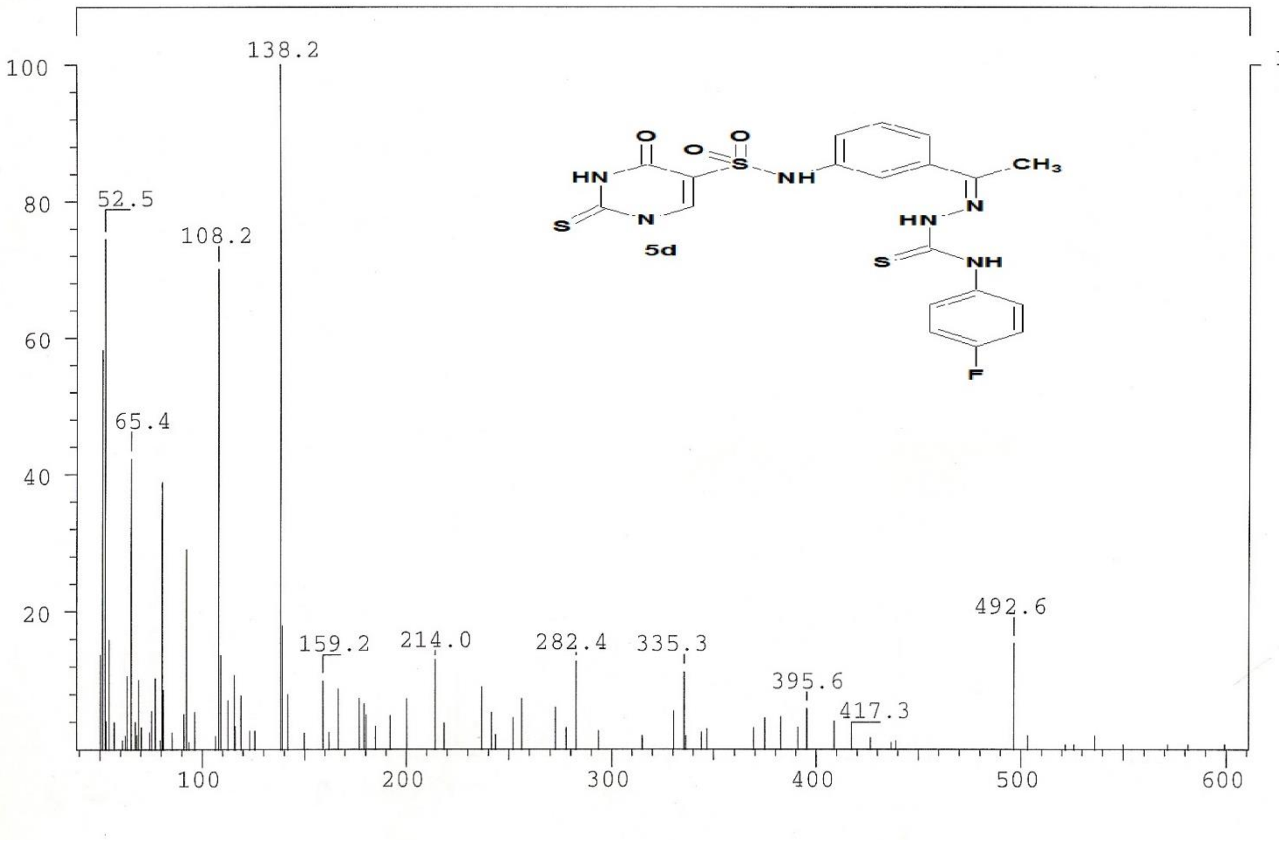

FigureS16. (EI mass) spectrum of compound 5d
